# Surgical challenges of simultaneous laparoscopic management of gastrointestinal stromal tumor and adrenocortical nodular hyperplasia in a 39-year-old female patient: a case report

**DOI:** 10.1093/jscr/rjae616

**Published:** 2024-10-03

**Authors:** Ali Gavrankapetanovic, Admir Bektesevic, Sanela Brzika, Nedim Hasic, Emina Letic, Ismar Rasic

**Affiliations:** Department of Surgery, General Hospital “Prim. dr. Abdulah Nakas”, Sarajevo, 71000, Bosnia and Herzegovina; Department of Surgery, General Hospital “Prim. dr. Abdulah Nakas”, Sarajevo, 71000, Bosnia and Herzegovina; Department of Surgery, General Hospital “Prim. dr. Abdulah Nakas”, Sarajevo, 71000, Bosnia and Herzegovina; Department of Surgery, General Hospital “Prim. dr. Abdulah Nakas”, Sarajevo, 71000, Bosnia and Herzegovina; Department of Surgery, General Hospital “Prim. dr. Abdulah Nakas”, Sarajevo, 71000, Bosnia and Herzegovina; Department of Surgery, General Hospital “Prim. dr. Abdulah Nakas”, Sarajevo, 71000, Bosnia and Herzegovina

**Keywords:** adrenocortical nodular hyperplasia, gastrointestinal stromal tumor, hyperaldosteronism, hypertension, surgical resection

## Abstract

Adrenocortical nodular hyperplasia and gastrointestinal stromal tumors are rare conditions, and their simultaneous occurrence in a single patient poses diagnostic and therapeutic challenges. Here, we present the case of a 39-year-old female patient who underwent surgical resection for concurrent adrenocortical nodular hyperplasia and GIST on the posterior part of the gastric fundus. The patient presented with symptoms of hyperaldosteronism and malignant hypertension, leading to the discovery of these two distinct tumors. Preoperative evaluation revealed normal laboratory findings and hormone levels, except for hyperaldosteronism and hypertension. The surgical intervention included left suprarenal gland removal and wedge resection of the gastric tumor. The patient experienced a successful outcome without intraoperative complications and remained normotensive during follow-up visits, with sustaining hormonal balance. This case underscores the importance of multidisciplinary collaboration and tailored surgical planning in managing complex neoplastic conditions.

## Introduction

Gastrointestinal stromal tumors (GISTs) are a rare form of neoplasm of the gastrointestinal (GI) tract that is associated with a high percentage rate of malignant alteration [1].

ANH is a condition that can cause sustained or paroxysmal hypertension, severe headaches, palpitations, and sweating due to hormone excess. If left untreated, they can be life-threatening [2]. In this case, we present a 39-year-old woman who was admitted to our hospital for surgical treatment of a large GIST and adrenal nodular hyperplasia. Preoperative evaluation revealed normal laboratory findings and hormone levels, except for hyperaldosteronism and hypertension. The surgical intervention included laparoscopic left suprarenal gland removal and wedge resection of the gastric tumor. During the follow-up visits, the patient had a sustaining hormonal balance with normotensive blood pressure and also reported withdrawal of headaches and stomach pains.

## Case report

A 39-year-old woman was admitted to our hospital for surgical treatment of a large GIST and Adrenal Nodular Hyperplasia. Previously she had gallbladder surgery and had suffered from pain in the epigastrium with an extension to her back. Also, she had secondary hypertension and severe headaches in the past few months. Two months before admission, the patient underwent a proximal endoscopy, an MRI, and a CT scan.

**Figure 1:**
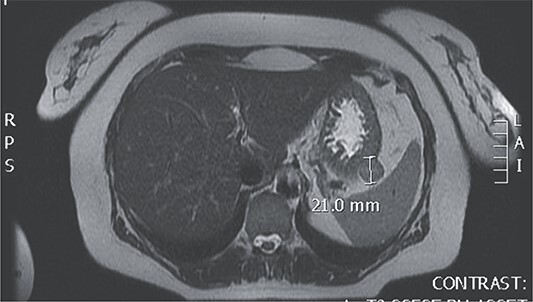
Magnetic resonance of GIST at the posterior of the fundus of the stomach.

Upon admission to our hospital, the patient had malignant hypertension and a persistent headache that couldn’t be mitigated with the use of analgesics. Laboratory tests showed elevated serum levels of aldosterone under stress (472 ng/dL) (reference range: 1.3–23.3 ng/dL) and elevated levels of aldosterone at rest (64 ng/dL) (reference range: 1.4–15.6 ng/dL), with all the other laboratory findings including serum levels of minerals, Hromangin A, urine levels of methanephrin in the reference range. An MRI finding showed a GIST tumor at the posterior of the fundus of the stomach (Fig. 1).

**Figure 2:**
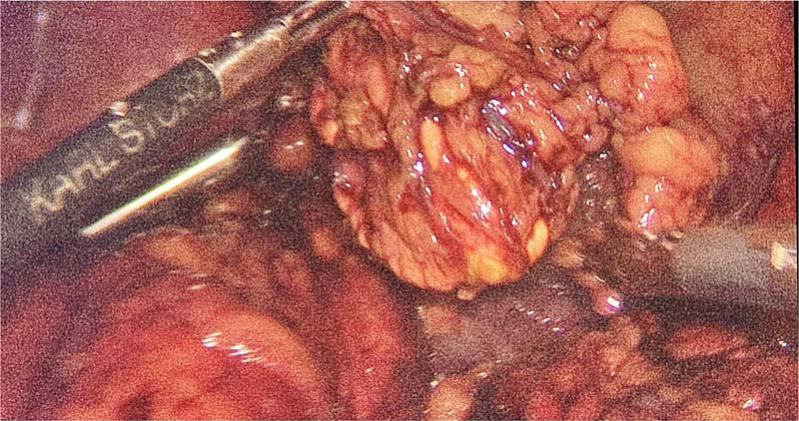
Intraoperative finding of the left suprarenal gland.

Intraoperatively, the location and resection of the left suprarenal gland (Fig. 2) was found located on the apex of left kidney underneath the body of the pancreas.

**Figure 3:**
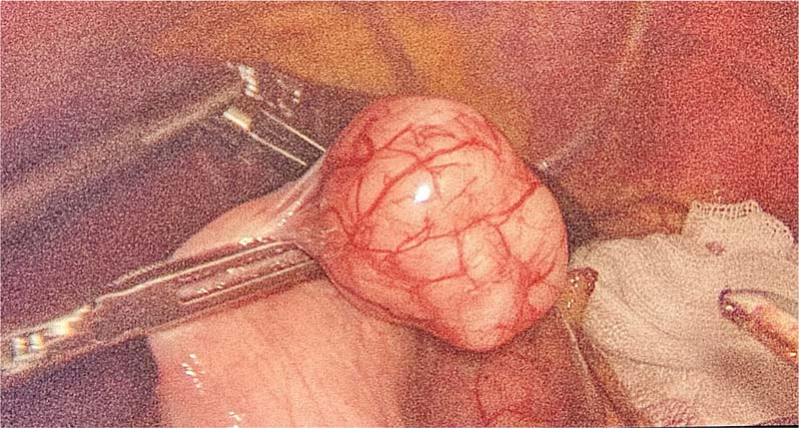
Intraoperative view of GIST.

**Figure 4:**
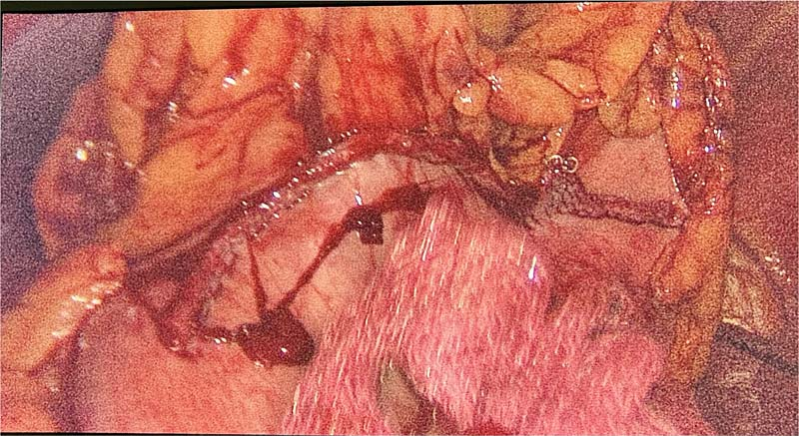
Intraoperative view of the stomache after wedge resection.

We had to first liberate the adhesions of the colon from a previous operation before gaining entry to the left colon. To do so, we made an incision in the White line of Toldt and dissected the right gastrocolic and splenocholic ligaments. This allowed us to access the retrocolic space and approach the left kidney. We identified the left renal vein and the left suprarenal gland and dissected the gland from the fatty tissue of the kidney. The gastrosplenic ligament was also liberated through the ligation of short gastric arteries. After separating the stomach from the posterior abdominal wall, we located the GIST (Fig. 3) on the posterior part of the fundus and removed it through wedge resection (Fig. 4).

After the surgery, she was closely monitored for any possible complications. There were no signs of intra-abdominal bleeding, high blood pressure, intra-abdominal infection, abscess, or delayed wound healing. As a result, the patient was discharged from the hospital on the 14th day following the surgery. During the follow-up, the patient had her blood pressure and hormone levels normalized. The follow-up results showed that the aldosterone at rest was 14.2 ng/dL. She also reported withdrawal of headaches and stomach pains. The pathological result came in as micro- and macronodular hyperplasia of the left suprarenal gland and GIST with a very low risk of progression.

## Discussion

GISTs are accounting for ~1%–2% of GI neoplasms [3]. On average, 18% of patients had an incidental diagnosis (ranging from 5% to 40%) [4].

The clinical symptoms of GISTs can vary greatly, depending on the size and location of the tumor [5]. Some common symptoms include abdominal pain, discomfort, fullness, early satiety, and a palpable mass, and sometimes GISTs can cause melena, hematemesis, dysphagia, and gastric outlet obstruction [6].

Primary pigmented nodular adrenocortical disease (PPNAD) and isolated micronodular adrenal disease (iMAD) fall under the micronodular types of adrenal cortex hyperplasias. These conditions are characterized by small nodules or lesions on the adrenal cortex. In macronodular form, the adrenal glands contain larger nodules or lesions compared to micronodular hyperplasias. While both micronodular and macronodular adrenal hyperplasias can cause Cushing syndrome due to excess cortisol production, they differ in the size and distribution of the nodules within the adrenal glands [7, 8].

The diagnosis of ANH relies on imaging tests, such as computed tomography and magnetic resonance imaging, of the adrenal glands and abdomen, which can help locate the tumor. Additional tests like 123I-metaiodobenzylguanidine scintigraphy and 18F-dihydroxyphenylalanine-positron emission tomography are also available [9].

Primary aldosteronism (PA) is a condition wherein there is an excessive production of aldosterone, which is not regulated by angiotensin II (Ang II), its normal chronic regulator. In the past, Conn’s syndrome was believed to be the most common cause of PA, found in subjects with aldosterone-producing adrenal adenomas [10, 11].

The occurrence of GIST and ANH at the same time is a rare and challenging clinical situation, especially in a young female patient. Managing multiple rare tumors concurrently requires a multidisciplinary approach involving surgical, medical, and oncological expertise. This case highlights the complexity of such situations.

Accurate diagnosis is paramount in guiding treatment decisions for patients with coexisting GIST and ANH. However, diagnosing both tumors simultaneously can be challenging due to their varied presentations and overlapping symptoms. Surgical management is the primary mode of treatment for both GIST and ANH. However, when there are two different tumors located close to each other, it presents a unique challenge for surgical planning and execution, and it is often better to remove both tumors simultaneously [2]. This approach minimizes the need for multiple surgeries and reduces perioperative risks. Managing anesthesia in patients with ANH requires careful attention to prevent sudden hypertensive crises and cardiovascular instability during surgery. In our case, we initiated preoperative alpha-adrenergic blockade to reduce the risk of hemodynamic fluctuations during surgery.

Despite the advancements in surgical techniques and adjuvant therapies, the prognosis for patients with concurrent GIST and ANH remains poor. This is mainly due to the possibility of tumor recurrence and metastasis. Therefore, conducting regular imaging studies and biochemical testing for long-term surveillance is vital.

Achieving the best possible outcomes for patients relies on accurate diagnosis, careful surgical planning, and diligent perioperative care. Additional research is necessary to understand the genetic and molecular mechanisms that drive the co-occurrence of these rare tumors and to develop targeted therapies tailored to meet individual patient needs.

## References

[1] Parab TM , DeRogatis MJ, Boaz AM, et al. Gastrointestinal stromal tumors: a comprehensive review. J Gastrointest Oncol 2019;10:144–54. 10.21037/jgo.2018.08.20. 30788170 PMC6351301

[2] Kim HJ , Choi GS, Park JS, et al. Simultaneous laparoscopic multi-organ resection combined with colorectal cancer: comparison with non-combined surgery. World J Gastroenterol 2012;18:806–13. 10.3748/wjg.v18.i8.806. 22371641 PMC3286144

[3] Beltran MA , Cruces KS. Primary tumors of jejunum and ileum as a cause of intestinal obstruction: a case control study. Int J Surg 2007;5:183–91. 10.1016/j.ijsu.2006.05.006. 17509501

[4] Soreide K , Sandvik OM, Soreide JA, et al. Global epidemiology of gastrointestinal stromal tumours (GIST): a systematic review of population-based cohort studies. Cancer Epidemiol 2016;40:39–46. 10.1016/j.canep.2015.10.031. 26618334

[5] Kersting S , Janot-Matuschek MS, Schnitzler C, et al. GIST: correlation of risk classifications and outcome. J Med Life 2022;15:932–43. 10.25122/jml-2021-0110. 36188659 PMC9514809

[6] Jaros D , Bozic B, Sebesta C. Gastrointestinale Stromatumoren (GIST) [Gastrointestinal stromal tumors (GIST)]. Wien Med Wochenschr. 2023;173:201–5. 10.1007/s10354-022-00965-8 36155864

[7] Berthon A , Bertherat J. Update of genetic and molecular causes of adrenocortical hyperplasias causing Cushing syndrome. Horm Metab Res 2020;52:598–606. 10.1055/a-1061-7349. 32097969

[8] Bourdeau I , Parisien-La Salle S, Lacroix A. Adrenocortical hyperplasia: a multifaceted disease. Best Pract Res Clin Endocrinol Metab 2020;34:101386. 10.1016/j.beem.2020.101386. 32115357

[9] Wang F , Liu J, Zhang R, et al. CT and MRI of adrenal gland pathologies. Quant Imaging Med Surg 2018;8:853–75. 10.21037/qims.2018.09.13. 30306064 PMC6177362

[10] Freel EM , Connell JM. Mechanisms of hypertension: the expanding role of aldosterone. J Am Soc Nephrol 2004;15:1993–2001. 10.1097/01.ASN.0000132473.50966.14. 15284285 PMC1283142

[11] Gordon R . Mineralocorticoid hypertension. Lancet 1994;344:240–3. 7913162 10.1016/s0140-6736(94)93003-1

